# Floral Scent Variation in the Heterostylous Species *Gelsemium sempervirens*

**DOI:** 10.3390/molecules24152818

**Published:** 2019-08-02

**Authors:** Bettie Obi Johnson, Annette M. Golonka, Austin Blackwell, Iver Vazquez, Nigel Wolfram

**Affiliations:** Division of Math, Science, Nursing, and Public Health, University of South Carolina Lancaster, 476 Hubbard Drive, Lancaster, SC 29720, USA

**Keywords:** *Gelsemium sempervirens*, Gelsemiaceae, yellow jessamine, Carolina Jessamine, flower, heterostyly, floral scent, volatile organic compounds, VOCs, benzenoid, SPME-GC-MS

## Abstract

*Gelsemium sempervirens* (L.) W.T. Aiton, a distylous woody vine of the family Gelsemiaceae, produces sweetly fragrant flowers that are known for the toxic alkaloids they contain. The composition of this plant’s floral scent has not previously been determined. In this study, the scent profiles of 74 flowers obtained from six different wild and cultivated populations of *G. sempervirens* were measured by solid phase microextraction-gas chromatography-mass spectrometry (SPME-GC-MS). There were 81 volatile organic compounds identified and characterized as benzenoids, terpenoids, fatty acid derivatives, and yeast associated compounds. The most abundant compound was benzaldehyde (23–80%) followed by ethanol (0.9–17%), benzyl benzoate (2–15%), 4-anisaldehyde (2–11%), (*Z*)-α-ocimene (0–34%), and α-farnesene (0.1–16%). The impacts of geographic location, population type (wild or cultivated), and style morph (L = long, S = short) on scent profile were investigated. The results showed no relationship between geographic location or population type and volatile organic compounds (VOC) profile, but did show a significant scent profile difference between L and S morphs based on non-metric multidimensional scaling (NMDS) using Bray-Curtis similarity indices. The L morphs contained higher amounts of benzenoids and the S morphs contained higher amounts of terpenoids in their scent profiles. The L morphs also produced a higher total abundance of scent compounds than the S morphs. This study represents the first floral scent determination of *G. sempervirens* finding significant variation in scent abundance and composition between style morphs.

## 1. Introduction

Flowering plants often produce attractants for pollinators in the form of scent [1,2,3,4], color [5], and nectar [6]. The scent profile of a flower may be due to petals, sepals, anthers, stigmas, and nectaries [7,8,9,10,11] and is composed of many different volatile organic compounds (VOCs). These VOCs are natural lipophilic products that have relatively low molecular weights (30–300 amu) and high vapor pressures, allowing easy detection at ambient temperatures [12]. Different parts of the plant emit VOCs, but the flowers typically emit the largest portion of scent from a plant [13]. Floral scent compounds promote cross-pollination and reproduction of flowers by attracting insect and other animal pollinators [14], who are rewarded with nectar and nourishment as pollinators help to distribute pollen amongst flowers. Over 1700 floral scent compounds [12] have been determined for a variety of plant species and are comprised of terpenoids (molecules derived from isoprene), benzenoids (molecules containing a benzene ring), fatty acid derivatives (aliphatic molecules with 1 to 25 carbon atoms), and yeast associated compounds (metabolic by-products such as ethanol), all with a variety of functional groups including alkanes, alkenes, aldehydes, ketones, alcohols, carboxylic acids, and esters [15]. The intensity and distribution of these scent compounds work together with visual and other cues to attract specific pollinators to a flower [14]. A typical floral bouquet is comprised of 20 to 60 compounds emitted between 1 pg and 30 µg per hour [15]. The compounds produced can vary between flower populations, style morphs, flower and plant tissues, time of day (light), temperature, and herbivory events (reviewed in [16]).

Although floral scent has been well characterized in several plant families [12], none of the scent profiles of flowering plants within Gelsemiaceae have been examined. *Gelsemium sempervirens* (L.) W.T. Aiton, commonly known as yellow jessamine or Carolina jessamine, is a toxic, perennial, distylous woody vine of the family Gelsemiaceae, which is only comprised of three genera [17,18]. *G. sempervirens* is indigenous to the Piedmont and coastal areas of the southeastern United States [19], and its flowers produce nectar and are pollinated by a variety of bee species [20,21]. This plant has been cultivated since the mid-17th century [19] and is known for its fragrant aroma, but also for the toxicity of its nectar, flowers, and other plant parts [19,22]. The medicinal and toxicological properties result from the presence of gelsemine and other alkaloids in plants from the genus *Gelsemium*, which have been widely studied and characterized [23,24]. The alkaloids in *G. sempervirens* act as deterrents for antagonists who consume nectar, flower petals, and reproductive structures without pollinating the plant [20]. Higher nectar gelsemine levels have been correlated with fewer visitations and reduced pollen transfer between flowers [20,21]. It has been suggested that *G. sempervirens* evolved to bloom in early spring, when there is little competition for pollinators from other flowering plants, in order to counteract its toxicity and ensure its reproductive success [25].

**Figure 1:**
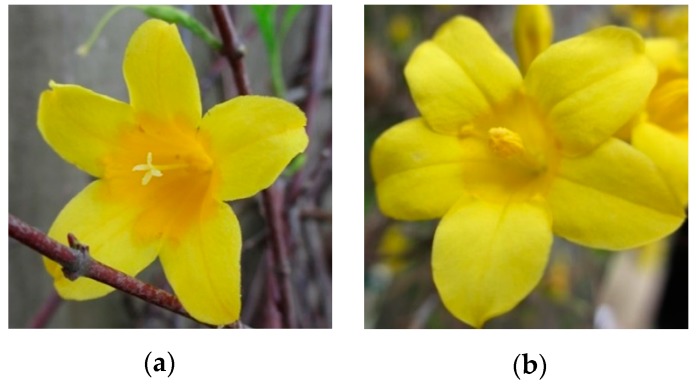
*Gelsemium sempervirens* flowers: (**a**) l-styled pin morph and (**b**) *S*-styled thrum morph.

The tubular shaped hermaphroditic flowers exist in two distinct forms that are self-incompatible within plant and style morph. The pin morph, also referred to as the L styled morph, has long styles with the stigmas protruding from the opening of the tubular flower and short stamens that remain within the floral tube (Figure 1a). The thrum morph, also referred to as the S styled morph, has short styles with long anthers protruding from the opening of the flower (Figure 1b) [26]. While it has been shown that flowers of the S morph are on average larger (S corollas are 11% longer and 26% wider than L corollas) [27,28] and contain on average higher levels of gelsemine than the L morphs [26], it is unknown whether floral scent varies between the L and S morphs. It can be hypothesized that the smaller sized L flowers would require a stronger, more attractive scent to attract flower visitors or to defend against florivory and larceny [27]. Only few studies have compared floral scent between style morphs in heterostylous angiosperms. While one study of two *Primula* heterostylous species found no significant differences in the scent strength or composition between L and S morphs [29], studies of several dioecious plants have found significant differences in the quantity and makeup of floral scent between male and female plants (reviewed in [30]).

The primary goal of this study was to elucidate the overall floral scent composition of the L and S flowers of *G. sempervirens* in populations located in the piedmont region of South Carolina. While the fragrant and sweet scent of this plant species has been noted in previous studies [19], to date it has not yet been chemically characterized. The secondary goal of this study was to investigate the variation in floral scent abundance and profile with geographic location, population type (wild or cultivated), and style morph (L or S). Significant differences in scent profile were expected between different populations based on a number of studies that have found differences in both emission rate and floral scent profile in several plant species from different geographic locations (reviewed in [31]). This variation is thought to be the result of pollinator-mediated selection as well as genetic drift. The information obtained in this study will provide a better understanding of floral scent chemistry and its contribution to plant-pollinator interactions in *G. sempervirens*.

## 2. Results

### 2.1. Overall Scent Profile

Within the six populations of *G. sempervirens* sampled (three cultivated and three wild populations), a total of 74 flowers were collected. From these 74 flowers, a total of 165 VOCs was detected, 81 of which were identified (Table 1). The detected compounds only include those produced by the flowers as the background compounds (scent of the environment and vial gas-off) were removed by comparison with a control air sample collected with each flower set. On average, each flower emitted 29 scent compounds. The identified scent compounds included 20 benzenoids, 18 terpenoids, 37 fatty acid derivatives, and six yeast associated compounds, all representing more than 98% of the total peak area for the detected VOCs in these flowers. The most common and abundant of these compounds are listed in Table 2.

**Figure 2:**
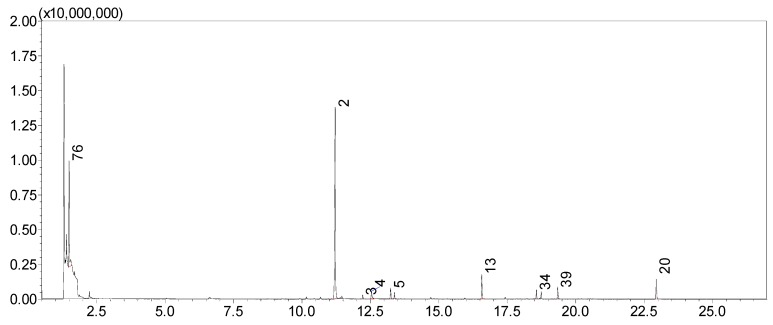
Total ion chromatogram of VOCs collected from an L flower in the W2 population. Numbers correspond to the chemical compounds in Table 2. Peaks for major compounds with amounts >1% are labeled.

A typical headspace total ion chromatogram (Figure 2) shows the predominant compound in this floral scent is benzaldehyde which was present in every flower sampled and averaged between 23% and 80% of the VOC peak areas. In addition to benzaldehyde, other benzenoids found in these flowers included benzyl benzoate (2–15%), 4-anisaldehyde (2–11%), acetophenone (0.4–6%), 4-methylanisole (0.2–4%), and benzyl alcohol (0.3–3.5%). Terpenoids were the next most abundant class of compounds and included (*Z*)-α-ocimene (0–34%), α-farnesene (0.1–16%), β-ionone (0–9%), dihydro-β-ionone (0–5%), α-ionone (0.03–3.1%), and α-pinene (0.01–4%). Fatty acid derivatives were present in smaller quantities and included 2-pentadecanone (0–12%), 2-tridecanone (0–4%), and 2-heptadecanone (0–3%). Ethanol (2–17%) was found in most of the floral scent profiles and is known to be associated with nectar inhabiting yeasts [32,33].

For all 74 flowers sampled for floral scent, the total peak areas ranged from 2.5 million to 117 million counts. These values were used to estimate floral scent strength of the flowers. As measured by total peak area, the floral scent strength emitted from L flowers was greater than the scent of S flowers (χ^2^ = 3.3, df = 1, *p* = 0.07). The L flowers had an average total peak area of 49.6 ± 4.9 million and the S flowers had an average total peak area of 36.8 ± 3.7 million. Conversely, there was no detectable difference in total peak area between the flowers within wild (43.3 ± 5.3 million) and cultivated (43.5 ± 3.8 million) populations (χ^2^ = 0.4, df = 1, *p* = 0.5).

### 2.2. Variation in Compound Classes

**Figure 3:**
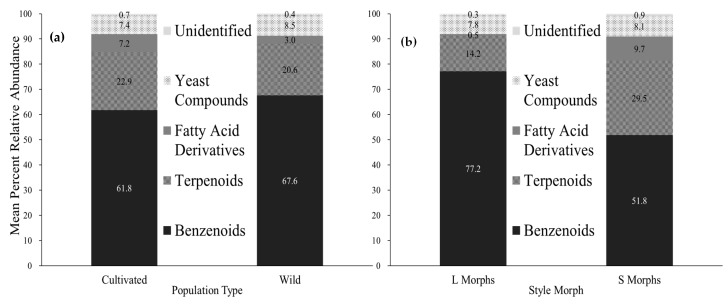
Mean percent relative abundance of compound classes for (**a**) cultivated (N = 35) and wild (N = 39) populations. (**b**) L (N = 38) and S (N = 36) styled flowers.

Grouping compounds together by class allowed an assessment of similarities and differences between the population types and style morphs (Figure 3). The mean benzenoid and terpenoid percentages were not significantly different between cultivated (61.8% benzenoids, 22.9% terpenoids) and wild (67.6% benzenoids, 20.6% terpenoids) populations (*p* > 0.5 for both compound classes) but these two compound groups were significantly different between L (77.2% benzenoids, 14.2% terpenoids) and S (51.8% benzenoids, 29.5% terpenoids) styled flowers (benzenoids: χ^2^ = 6.1, df = 1, *p* = 0.014; terpenoids: χ^2^ = 7.1, df = 1, *p* = 0.008). The fatty acid derivatives varied between wild (2.9%) and cultivated (7.2%) populations (χ^2^ = 8.7, df = 1, *p* = 0.003) as well as between L (0.5%) and S (9.7%) styled morphs (χ^2^ = 8.9, df = 1, *p* = 0.003). The yeast associated scent compounds ranged from 7 to 8% with no significant differences between population types or style morphs (*p* > 0.4 for both population type and style type)**.**

### 2.3. Variation in Complete Scent Profiles

**Figure 4:**
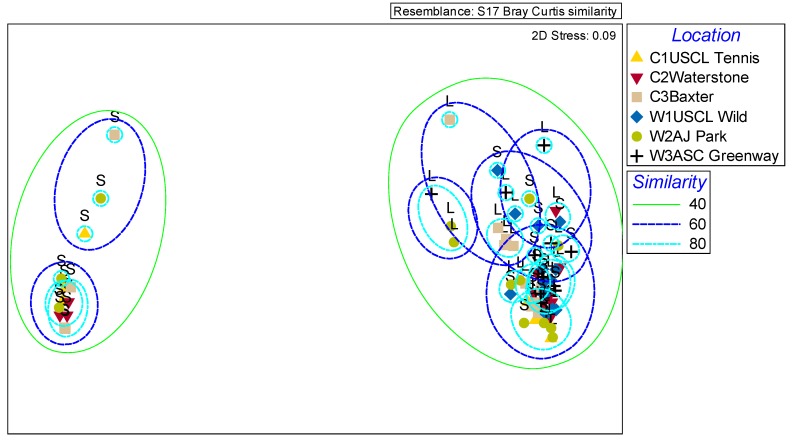
Non-metric multidimensional scaling plot of the relative proportions of all floral volatiles emitted by L and S flowered morphs based on the Bray-Curtis dissimilarity index (stress = 0.09). Population locations are indicated by different symbols. The lines around the two groups indicate similarity percentages based on cluster analysis comparing the overall scent profiles.

A nested PERMANOVA of all the VOCs within the scent profiles indicated there was no significant difference between the scent profiles of wild and cultivated population types (Pseudo-F = 2.1, df = 1, *p* = 0.3). A non-metric multidimensional scaling (NMDS) plot (Figure 4) using a cluster analysis of the Bray-Curtis dissimilarity indices based on the relative amounts of each VOC also showed no correlation between population type (wild or cultivated) and complete scent profile. However, there were significant differences in the overall scent profile among geographic locations (C1, C2, C3, W1, W2, W3) nested within population type (Pseudo-F = 2.2, df = 4, *p* = 0.022). Additionally, significant differences were found when style type (L, S) was nested within population location nested within wild and cultivated population (Pseudo-F = 3.6, df = 6, *p* = 0.0001).

The cluster analysis and NMDS plot supports the significant difference between style morph (L, S) for complete scent profile (Figure 4). In the NMDS plot, there is a clear separation between the scent profiles of some of the S styled morphs which are clustered together without any L morphs with 40% similarity. All of the L styled morphs along with some of the S styled morphs are clustered together with 40% similarity for complete scent profile. These results indicate that many of the S styled flowers have very dissimilar scent profiles to the L styled flowers, regardless of which population the flowers were sampled from. Of the 81 identified compounds, 26 of the benzenoids, terpenoids, and fatty acid derivative compounds were significantly different for relative amounts across L and S morphs (see compounds with an * in Table 1). All 8 benzenoid compounds that were significantly different across the style morphs were higher in the L flowers versus the S flowers. There was more variability in the terpenoid and fatty acid derivative compounds with only half of the significantly different terpenoid compounds present in higher amounts in the L flowers, and only 10% of the significantly different fatty acid derivatives present in higher amounts in the L flowers versus the S flowers. These results reiterate the compound class differences seen earlier in Figure 3.

## 3. Discussion

The compounds emanating from flowers of *G. sempervirens* provide the sweet and fragrant aroma that is characteristic of the species, and several of the compounds are commonly found in angiosperms [12]. The predominant compound in *G. sempervirens* floral scent was benzaldehyde, present in all flowers sampled ranging from 23% to 80% of the overall floral scent profile. Benzaldehyde is a benzenoid compound with a sweet almond-like odor [34]. The other benzenoids (benzyl benzoate, 4-anisaldehyde, acetophenone, 4-methylanisole, and benzyl alcohol) all have sweet odors of varying strengths [34] and ranged in concentration in the floral scent profile of *G. sempervirens*. Terpenoids were the next most abundant class of compounds with wide concentration ranges, with (*Z*)-α-ocimene and α-farnesene as the most common terpenoids. The terpenoids add sweet, floral, woody scents to the floral fragrance [34]. Fatty acid derivatives, also known as green leaf volatiles, were present in smaller quantities and may have been formed by the flower’s sepals or the small amount of stem needed to ensure the flower remained healthy within the vial during the sampling period. Research into what part of the flower produced the VOCs may help determine if the presence of these green leaf volatiles is actually due to the petals, stamens, stigmas, or sepals/stem. Some compounds associated with yeasts [32,33], such as ethanol, were also found in most of the floral scent profiles, indicating the possible presence of microbes either within the nectar or on the flower parts of this plant. Future research is planned to explore the presence of nectarivorous microbes on *G. sempervirens*.

The floral scent compounds produced by this plant makes it ideal for both generalists and specialist pollinators. The floral scent of most bee-pollinated plants contains terpenoids with small amounts of benzenoids and fatty acid derivatives; however, there are some bee-pollinated plants whose floral scents contain mostly benzenoids including *Cucurbita, Pyrola, Petunia, Trifolium pratense, Couratari, Lecythis, Antirrhium,* and *Tritoniopsis* [35]. In bee behavioral studies, certain benzenoids including some of those detected in *G. sempervirens* (4-anisaldehyde, benzyl acetate, benzyl alcohol, and 1,4-dimethoxybenzene) elicited positive behavioral responses in *Apis mellifera* and other bee species [36]. Diurnal butterflies pollinate flowers with similar scent compounds found in *G. sempervirens* including benzaldehyde, benzyl alcohol, β-ocimene, and cis-3-hexenyl acetate [35]. Diurnal moths are attracted to a variety of compounds depending on the plant species, but they tend to be attracted to similar VOCs as butterflies, including benzaldehyde, β-ocimene, and cis-3-hexenyl acetate [35]. The hovering moths (Sphingidae) are known to be attracted to a wide variety of compounds, again depending on the plant species, including methyl benzoate, benzyl acetate, β-ocimene, farnesene, and cis-3-hexenyl acetate [35]. Most of the compounds listed above were present in *G. sempervirens*, indicating perhaps a very generalist floral scent profile to accommodate a variety of pollinators. Previous research has found that *G. sempervirens* is pollinated by various species of bees including Apidae (*Bombus bimaculatus, Apis mellifera,* and *Habropoda laboriosa*) and Megachilidae (*Osmia lignaria*) [26,37]. Observations of plants near South Carolina population W1 indicated that butterfly and diurnal sphingids (specifically, *Amphion floridensis*) also visit the flowers (Golonka, unpublished data). Future research is needed to determine how floral scent varies temporally, if *G. sempervirens* is a predominantly diurnal- or nocturnal-pollinated plant species, and which pollinators contribute to the seed set.

The significant difference in overall scent profile among geographic locations indicates there were population differences independent of whether or not the plants within the population were considered cultivated or wild. Both cultivated and wild populations that were sampled from six different locations in SC had similar relative abundances of benzenoid and terpenoid compounds within the scent profiles of their flowers. However, the fatty acid derivatives were more prevalent in the cultivated populations than in the wild populations (Figure 3). An analysis of all the compounds also indicated no significant difference in compound presence between cultivated and wild populations. However, there were significant differences in the overall scent profile among geographic locations (C1, C2, C3, W1, W2, W3) nested within population types, indicating the possibility of within site differences. More research needs to occur to determine if there are maternal effects, ecological effects (soil pH, temperature, moisture, etc), or geographical differences across the populations. Significant differences in floral traits of *G. sempervirens* collected from wild and cultivated sites were found by Irwin et al. [28], with wider corolla tubes in wild sites and longer corolla tubes in cultivated sites. Corolla tube length and width were also found to be positively correlated with the age of the cultivated site with older sites having longer corolla tubes. The age of the cultivated site in this study was not taken into account, but this is an aspect that can be examined in future studies. Irwin et al. [28] also found several floral traits were phenotypically and genotypically correlated, with cultivated and wild plants producing similar results in a common garden experiment as listed above. Other studies have indicated that scent profile does vary based on temperature [38,39], light [39,40], time [7], pollination status [7], and geographic location [7].

L styled flowers were significantly stronger scented than S styled flowers. The stronger scent of L styled flowers is consistent with the hypothesis that the smaller flowers will produce a stronger scent to attract pollinators or to defend against florivory and larceny, but further research will be necessary to distinguish between these options [27]. There were also significant differences between compound classes across the style morphs of this plant. Benzenoids represent a larger proportion of the overall scent in L flowers while terpenoids and fatty acid derivatives represented a larger proportion of the overall scent profile of S flowers. Yeast associated scent compounds were equally likely in both population types and style morphs. These differences held up even when the overall scent profile was analyzed and style morph was nested within geographic location nested within population type, indicating strong differences in style morphs for complete scent profile (Figure 4). This is particularly clear in the NMDS analysis where the plot indicates a clear separation between the scent profiles of some of the S flowers and the majority of the L flowers. These results indicate that many of the S flowers have very dissimilar scent profiles to the L flowers, regardless of which population the flowers were sampled from. Of the 81 identified compounds, 26 of the benzenoids, terpenoids, and fatty acid derivative compounds were significantly different for relative amounts across L and S flowers (see compounds with an * in Table 1). All 8 benzenoid compounds that were significantly different across the style morphs were higher in the L flowers than the S flowers. There was more variability in the terpenoid and fatty acid derivative compounds with only half of the significantly different terpenoid compounds present in higher amounts in the L flowers, and only 10% of the significantly different fatty acid derivatives present in higher amounts in the L flowers versus the S flowers.

A previous investigation of floral odor variation in heterostylous species *Primula elatior* and *P. farinosa* found significant differences in the floral bouquets of each species but found no significant differences between the L and S morphs of each species [29]. It was reasoned that because heterostylous flowers cannot self-pollinate, they rely on pollinators visiting both floral morphs equally. Selection against divergence in traits of the two morphs promotes constancy and provides a reproductive advantage for that species. However, studies on dioecious species have found significant differences in scent emission rate and scent composition between male and female plants [30]. The measured difference in floral scent abundance and VOC profile in the current study represents a unique finding relative to previous heterostyly studies. Given the fact that the L morphs are on average smaller than S morphs [27], it seems reasonable that floral scent would in turn also differ in *G. sempervirens*. The L morph flowers present a stronger attractive scent in terms of total scent compounds produced and a higher proportion of benzenoids, which may be attracting more visitors to these flowers. Irwin and Adler [26] found that L morphs received twice as much pollen as S morphs, which was partially explained by the higher level of gelsemine in S morphs. The current study indicates that floral scent may have also been a factor. More research needs to be conducted to determine if the stronger scent of the L morphs contributes to differential pollinator visitation, and thus pollen deposition.

## 4. Materials and Methods

### 4.1. Study Sites and Flower Collection

A total of 74 flowers of *G. sempervirens* (yellow jessamine/Carolina jessamine; Gelsemiaceae) were collected in March and April 2014 from 6 different populations, 3 cultivated and 3 wild, within a 51 km range in the piedmont region of South Carolina (Table 3). For each population, 6–8 flowers of each morph (L and S) were sampled (except for population C1 with 3 L flowers).

Each of the flowers was collected in 15-mL sterilized clear glass vials with PTFE/silicone septum caps. Six flowers and a control sample were collected each day between 7:00 and 9:00 am. At the collection site, 250 µL of sterile water was added to each sterilized vial. Wearing nitrile gloves, a fully opened flower with anthers visible was removed from the vine and placed into the vial using small forceps, with the open side of the flower pointed up.

The control sample contained 250 µL of sterile water and air from approximately 3 m away from the sampling location and was used to control for scent of the environment (i.e., background noise) and gas-off compounds from vials.

### 4.2. SPME-GC-MS Analysis of Flowers and Standard Compounds

Flower and control sample vials were taken back to the lab for static SPME-GC-MS analysis. Flowers were equilibrated in the vials for a minimum of 3 h with times ranging from 3 to 10 h depending on transport from site and wait times for SPME-GC-MS analysis. A SPME fiber coated with divinylbenzene/carboxen/polydimethylsiloxane (50/30 µm DVB/CAR/PDM, Supelco part #57348-U) was used to collect volatile scent compounds from the headspace above each flower, as done in previous studies [41,42]. This fiber coating phase was chosen because it has been found to absorb a wide range of VOCs (polar and nonpolar) at relatively low levels. The headspace VOC gases sampled by SPME were thermally desorbed and analyzed by GC-MS between 2.5 and 10 h after flower collection.

Prior to headspace collection, the fiber was thermally conditioned at 270 °C in the GC-MS injection port for 1 h. A Shimadzu QP 2010S GC-MS system was used with a 30 m 5% phenyl methyl silicone column (SHR5XLB, 30 m × 0.25 mm × 0.25 μm). A blank fiber injection was run to verify that no contaminants would bleed from the fiber during sample runs.

The SPME fiber was placed into the headspace of each vial (control and flower samples) for 25 min at room temperature to collect volatile scent compounds. The SPME fiber was then thermally desorbed at 250 °C in the injection port with a 4.5 mm sampling depth, using splitless injection mode for 0.5 min of sampling time. A temperature ramp was used in the GC-MS as follows: 35 °C for 5 min, 10 °C per minute to 250 °C for 0.50 min. The total run time was 27.0 min. During the run, the mass spectrometer was scanned over a mass range of 30.00 to 300.00 *m*/*z* units. The SPME fiber was left in the injection port for the entire run to ensure complete removal of volatile compounds from the fiber to prevent carryover between injections. The effectiveness of this cleansing method for the SPME fiber was demonstrated by injecting blank fibers after sample runs, showing little or no detectable compounds.

Compounds were identified using Wiley and National Institute of Standards and Technology mass spectral libraries (containing more than 120,000 reference mass spectra), and then verified by analyzing pure standard compounds. Standard compounds were analyzed from 250 µL of a 0.01% dilution of each standard in a 15-mL SPME vial. 1 µL of each standard was diluted to a total volume of 100 µL with sterile water in a plastic 1.5 mL micro-tube to make a 1% solution. 2.5 µL of this 1% solution was then added to 247.5 µL of sterile water in a 15-mL SPME vial to prepare the 0.01% standard solution. Standard solutions were prepared to contain between 1 and 6 standard compounds. Solutions were analyzed by exposing the SPME fiber to the headspace above the solution for 1 min at room temperature, and injecting the fiber into the GC-MS as described for the flower samples. Matching retention times and mass spectral ions confirmed the identification of each floral scent compound. Kovats retention indices (R.I.) were determined with hydrocarbon standards as described by Raguso [43]. Compounds that were not available to purchase as standards were confirmed by comparing the observed Kovats retention indices with literature values. Unidentified compounds were present in low concentrations (typically < 0.1%).

Compounds were quantified by integrating all chromatographic peaks and removing peaks present in the control samples (<10:1 sample:control peak area ratio). Relative amounts of each compound were estimated by dividing the peak area of each compound by the sum of all peak areas for a given sample and multiplying by 100. The strength of each flower’s scent was quantified by calculating the total peak area from each flower’s chromatogram.

### 4.3. Statistical Analysis

The chemical composition of all floral volatiles from L and S flowers, the six populations, and the two population types were analyzed using either PRIMER v6 with the PERMANOVA+ add-on package [44] or SAS [45]. Univariate analyses (PROC UNIVARIATE, [44]) indicated most of the data were non-normally distributed; therefore, multivariate analyses (NMDS, Cluster, and PERMANOVAs; [46]) were used to compare the overall scent profile of each sample along with non-parametric Wilcoxon and Kruskal-Wallis tests (NPAR1WAY, [45]) to compare relative percent areas of total peak area of VOCs and compound groups across sample types. A comparison of non-parametric (NPAR1WAY, [45]) analyses with one-way analyses of variances (TTEST, [45]) indicated very little difference in the results; therefore, the more conservative non-parametric results are presented.

Relative amounts of each compound were used to conduct non-metric multidimensional scaling (NMDS) analyses [47,48]. Relative amounts of each compound were used to calculate a similarity matrix between each sample type (style morphs [L vs. S], geographic locations [1, 2…6], population types [C vs. W]) using the Bray-Curtis similarity index. An iterative process was then applied to create a best-fit set of axes to represent scent profile similarity between sample types with close proximity in space indicating greater similarity in scent profiles between samples and greater distances representing more dissimilarity between samples [44,48,49]. A cluster analysis was performed on the similarity indices to determine at what level the sample types clustered together and results were superimposed on the MDS plots using PRIMER v6 [44].

As the data were not normal and did not fit the assumptions of a multivariate ANOVA (MANOVA), an analogous process, permutational multivariate analysis of variance (PERMANOVA), was used to analyze the scent profiles of samples [48,50]. Using the Bray-Curtis similarity indices, a series of PERMANOVAs were performed on different data sets based on 9999 permutations. PERMANOVAs use similarity/dissimilarity resemblance indices to calculate pseudo-F and permutation-based P-values to test for the response of variables (i.e., VOCs) to one or more factors (i.e., population type, geographic location, and style morph). A nested PERMANOVA was performed on the complete floral scent indices to determine if there were differences in style type (L vs. S) nested within population location nested within population type (C vs. W).

## 5. Conclusions

The floral scent profile of *G. sempervirens* was found to be dominated by benzenoids (52–77%), mostly benzaldehyde, with smaller amounts of benzyl benzoate, 4-anisaldehyde, acetophenone, 4-methylanisole, and benzyl alcohol. The scent profile also contained smaller quantities of terpenoids (14–30%) including (*Z*)-α-ocimene, α-farnesene, β-ionone, dihydro-β-ionone, and α-ionone. Fatty acid derivatives were also detected and included 2-pentadecanone, 2-tridecanone, and 2-heptadecanone. Yeast associated compounds such as ethanol were detected in most flowers. Significant differences were found in total scent, compound class distribution, and complete scent profiles between the L and S styled morphs of this species. On average, the L flowers had a stronger scent and higher levels of benzenoids than the S flowers. Future studies will be aimed at determining other factors impacting *G. sempervirens*’ scent including time of day, life cycle stage, and ambient temperature/humidity levels.

## Figures and Tables

**Table 1:** Mean (s.e.) relative amounts of volatile organic compounds (VOCs) (≥0.01%) in L and S flower morphs from three cultivated (C1, C2, C3) and three wild (W1, W2, W3) populations of *G. sempervirens*. All scent compounds, except in brackets, were verified using standards. Unverified compounds were identified with >90% matches to library spectra and literature R.I. values. Compounds with significantly different relative amounts in L verses S flowers (*p* < 0.05) are indicated with an asterisk.

	Compound	R.I.	C1		C2		C3		W1		W2		W3	
			L (N = 3)	S (N = 6)	L (N = 6)	S (N = 6)	L (N = 8)	S (N = 6)	L (N = 8)	S (N = 6)	L (N = 7)	S (N = 6)	L (N = 6)	S (N = 6)
***Benzenoids***			***93 (3)***	***49 (21)***	***79 (5)***	***48 (19)***	***80 (5)***	***31 (17)***	***83 (3)***	***69 (7)***	***72 (7)***	***41 (18)***	***62 (10)***	***73 (4)***
1	Benzene	660	0.02 (0.02)	0.02 (0.02)	0.011 (0.004)	0.01 (0.01)	0.06 (0.02)	0.02 (0.01)	0.02 (0.01)	0.02 (0.02)	-	0.03 (0.02)	0.02 (0.01)	0.03 (0.02)
2	Benzaldehyde *	995	80 (6)	37 (16)	58 (5)	31 (13)	46 (7)	23 (14)	59 (6)	48 (7)	57 (8)	32 (15)	44 (9)	51 (4)
3	4-Methylanisole	1048	0.2 (0.1)	0.7 (0.4)	0.4 (0.1)	1.4 (0.7)	3.4 (0.7)	0.3 (0.2)	4 (1)	3.1 (0.8)	0.7 (0.1)	0.5 (0.3)	1.0 (0.2)	1.6 (0.4)
4	Benzyl alcohol *	1064	3.2 (0.8)	2 (1)	0.3 (0.1)	1.0 (0.5)	3.5 (0.7)	0.5 (0.3)	0.9 (0.5)	1.5 (0.7)	3.1 (0.9)	0.3 (0.1)	0.9 (0.4)	2 (1)
5	Acetophenone	1100	0.39 (0.08)	2.3 (0.6)	1.6 (0.6)	2.3 (0.8)	6 (1)	2.3 (0.4)	1.0 (0.5)	0.8 (0.4)	1.5 (0.3)	1.1 (0.3)	3.6 (0.9)	6 (1)
6	Methyl benzoate	1123	-	-	0.01 (0.01)	-	-	-	-	-	0.01 (0.01)	-	-	0.03 (0.03)
7	Benzyl acetate *	1190	0.03 (0.03)	0.02 (0.01)	0.3 (0.1)	0.08 (0.04)	0.37 (0.07)	0.04 (0.02)	0.11 (0.03)	0.15 (0.07)	0.25 (0.07)	0.06 (0.04)	0.16 (0.08)	0.09 (0.06)
8	Ethyl benzoate *	1197	-	-	0.04 (0.03)	-	0.06 (0.02)	0.01 (0.01)	-	0.01 (0.01)	0.16 (0.09)	0.02 (0.02)	0.07 (0.07)	0.09 (0.09)
9	1,4-Dimethoxybenzene *	1199	0.3 (0.1)	0.01 (0.01)	0.01 (0.01)	0.03 (0.02)	0.17 (0.08)	-	0.3 (0.1)	0.01 (0.01)	-	0.02 (0.02)	0.08 (0.06)	0.01 (0.01)
10	2`-Hydroxyacetophenone	1206	-	0.2 (0.1)	0.26 (0.07)	0.3 (0.2)	0.7 (0.2)	0.4 (0.1)	0.10 (0.04)	0.18 (0.05)	0.05 (0.02)	0.12 (0.04)	0.17 (0.08)	0.6 (0.2)
11	2-Methoxy-4-methylphenol	1223	-	0.01 (0.01)	-	0.04 (0.03)	0.08 (0.02)	-	-	-	-	0.01 (0.01)	0.03 (0.03)	0.01 (0.01)
12	Estragole	1230	0.09 (0.05)	0.02 (0.02)	0.03 (0.01)	0.10 (0.08)	0.29 (0.07)	0.01 (0.01)	0.10 (0.04)	0.12 (0.06)	0.01 (0.01)	0.03 (0.03)	0.04 (0.03)	0.10 (0.05)
13	4-Anisaldehyde *	1313	3.8 (0.1)	4 (2)	11 (1)	3 (2)	5 (1)	2 (1)	8 (1)	5 (1)	3.1 (0.6)	3 (2)	5 (2)	4.9 (0.8)
14	Isobutyl benzoate	1359	-	-	-	-	-	0.01 (0.01)	0.02 (0.01)	-	0.01 (0.01)	-	0.04 (0.02)	0.03 (0.01)
15	Benzyl butyrate *	1373	-	0.02 (0.01)	0.24 (0.08)	0.10 (0.06)	0.17 (0.04)	0.07 (0.05)	0.3 (0.2)	0.15 (0.03)	0.20 (0.07)	0.02 (0.01)	0.05 (0.02)	0.11 (0.07)
16	Butyl benzoate	1403	0.02 (0.01)	0.03 (0.01)	0.21 (0.03)	0.05 (0.03)	0.02 (0.01)	0.03 (0.02)	0.13 (0.05)	0.17 (0.04)	0.08 (0.01)	0.05 (0.03)	0.04 (0.02)	0.13 (0.04)
17	Benzyl tiglate	1534	0.09 (0.05)	0.04 (0.02)	0.01 (0.01)	0.09 (0.06)	0.10 (0.06)	0.03 (0.02)	0.01 (0.01)	0.02 (0.02)	0.02 (0.01)	0.05 (0.04)	0.01 (0.01)	0.05 (0.02)
18	(*Z*)-3-Hexenyl benzoate	1605	0.04 (0.01)	0.02 (0.01)	0.15 (0.04)	0.2 (0.1)	0.06 (0.02)	0.02 (0.01)	0.09 (0.03)	0.17 (0.06)	0.07 (0.04)	0.05 (0.04)	0.16 (0.05)	0.4 (0.2)
19	Hexyl benzoate	1612	-	0.01 (0.01)	0.05 (0.01)	0.06 (0.03)	0.02 (0.01)	0.02 (0.01)	0.05 (0.02)	0.04 (0.01)	0.04 (0.01)	0.03 (0.03)	0.02 (0.01)	0.05 (0.03)
20	Benzyl benzoate *	1823	5 (1)	3 (2)	6 (1)	8 (4)	15 (2)	2 (2)	8 (1)	9 (2)	5.7 (0.8)	3 (2)	6 (2)	5.8 (0.7)
***Terpenoids***			***3 (1)***	***26 (11)***	***20 (5)***	***31 (11)***	***9 (3)***	***43 (13)***	***14 (2)***	***24 (6)***	***10 (5)***	***30 (10)***	***26 (8)***	***24 (4)***
21	α-Pinene	936	0.4 (0.2)	0.25 (0.08)	0.01 (0.01)	0.8 (0.2)	4 (2)	0.29 (0.09)	0.15 (0.04)	0.18 (0.05)	0.11 (0.04)	0.06 (0.05)	0.2 (0.2)	0.04 (0.03)
22	[β-Phellandrene]	980	-	0.02 (0.01)	-	-	-	0.17 (0.09)	-	-	0.01 (0.01)	0.01 (0.01)	0.05 (0.05)	-
23	β-Pinene	982	0.13 (0.05)	0.02 (0.01)	-	0.24 (0.04)	2 (1)	0.09 (0.03)	0.01 (0.01)	-	0.03 (0.01)	-	0.05 (0.02)	0.02 (0.01)
24	Myrcene	994	-	-	-	-	-	0.05 (0.03)	-	-	-	0.07 (0.04)	-	-
25	6-Methyl-5-hepten-2-one	1009	0.1 (0.1)	0.05 (0.05)	0.47 (0.06)	1.2 (0.4)	0.21 (0.09)	0.3 (0.2)	0.4 (0.2)	1.0 (0.4)	0.5 (0.2)	0.5 (0.2)	0.35 (0.08)	0.7 (0.1)
26	1,8-Cineole *	1044	-	0.1 (0.1)	0.01 (0.01)	-	-	0.5 (0.3)	-	-	-	0.07 (0.04)	-	0.01 (0.01)
27	Limonene *	1045	-	-	0.03 (0.01)	0.1 (0.1)	-	-	0.05 (0.02)	-	-	-	0.02 (0.02)	-
28	(*E*)-β-Ocimene *	1046	0.08 (0.08)	0.13 (0.08)	-	0.3 (0.1)	-	0.3 (0.1)	-	-	-	0.08 (0.05)	-	-
29	(*Z*)-α-Ocimene *	1057	-	21 (9)	0.08 (0.03)	24 (11)	-	34 (13)	0.02 (0.01)	-	-	18 (9)	0.02 (0.01)	0.07 (0.01)
30	[Ionene]	1366	-	-	0.07 (0.05)	-	-	0.01 (0.01)	-	0.7 (0.2)	0.06 (0.04)	0.04 (0.04)	0.2 (0.2)	0.05 (0.04)
31	(E)-β-Caryophyllene *	1449	-	0.14 (0.06)	-	0.4 (0.1)	-	0.3 (0.2)	-	-	0.01 (0.01)	0.14 (0.06)	0.03 (0.03)	-
32	Dihydro-α-ionone	1450	-	-	0.8 (0.3)	-	0.3 (0.1)	0.05 (0.05)	0.02 (0.02)	0.4 (0.2)	0.08 (0.06)	-	0.3 (0.3)	0.6 (0.3)
33	Geranyl acetone	1451	-	-	-	0.12 (0.05)	-	0.01 (0.01)	0.03 (0.01)	0.03 (0.02)	0.04 (0.03)	0.03 (0.03)	0.3 (0.2)	0.03 (0.02)
34	α-Ionone *	1460	0.2 (0.1)	0.03 (0.02)	3.0 (0.8)	1.0 (0.6)	0.5 (0.3)	0.6 (0.4)	2.6 (0.8)	3.1 (0.8)	1.4 (0.5)	2 (1)	2.5 (0.9)	1.3 (0.5)
35	Dihydro-β-ionone *	1467	-	3 (2)	2 (1)	0.8 (0.5)	0.04 (0.03)	4 (2)	0.5 (0.3)	3 (1)	0.4 (0.2)	5 (2)	2 (1)	1.4 (0.7)
36	Neryl acetone	1470	1.1 (0.4)	0.8 (0.5)	0.09 (0.09)	1.5 (0.8)	0.14 (0.08)	-	1.1 (0.4)	0.4 (0.2)	0.8 (0.7)	1.0 (0.9)	1.3 (0.7)	0.5 (0.3)
37	[(Z)-α-Bergamotene]	1500	-	-	0.08 (0.01)	0.02 (0.02)	-	0.01 (0.01)	0.04 (0.02)	0.06 (0.05)	0.04 (0.03)	-	0.12 (0.05)	0.15 (0.04)
38	α-Farnesene *	1517	0.6 (0.2)	0.3 (0.1)	4 (2)	0.4 (0.2)	1.3 (0.5)	0.4 (0.2)	8 (2)	7 (5)	5 (4)	0.1 (0.1)	11 (4)	16 (5)
39	β-Ionone	1519	-	-	9 (4)	-	0.2 (0.1)	2 (2)	1 (1)	8 (4)	1.2 (0.7)	3 (3)	7 (7)	3 (3)
***Fatty Acid Derivatives***			***0.37 (0.06)***	***14 (6)***	***0.19 (0.04)***	***17 (7)***	***1.0 (0.3)***	***9 (4)***	***0.5 (0.2)***	***0.7 (0.5)***	***0.10 (0.06)***	***17 (8)***	***0.5 (0.1)***	***0.5 (0.3)***
40	Tert-butyl ethyl ether *	611	-	-	-	-	0.2 (0.1)	-	-	-	-	-	0.12 (0.07)	-
41	1-Butanol	674	-	-	-	-	-	-	0.03 (0.03)	-	-	-	-	-
42	Heptane	700	-	0.03 (0.03)	-	-	-	-	-	0.03 (0.03)	-	-	-	-
43	2-Pentanone	706	-	-	-	0.05 (0.05)	-	-	-	-	-	-	-	-
44	3-Pentanone	716	-	-	-	0.08 (0.05)	-	-	-	-	-	-	-	-
45	4-Methyl-2-pentanone	759	-	-	-	0.09 (0.09)	-	-	-	-	-	-	-	-
46	2-Methyl-2-butenal	769	0.07 (0.05)	-	-	-	-	0.04 (0.04)	-	-	0.01 (0.01)	0.2 (0.1)	-	-
47	Octane	800	-	0.01 (0.01)	-	0.06 (0.04)	-	-	0.01 (0.01)	0.1 (0.1)	-	0.03 (0.03)	-	-
48	4-Methyl-3-penten-2-one	824	-	-	-	0.15 (0.08)	-	-	-	0.01 (0.01)	0.02 (0.01)	0.02 (0.02)	-	-
49	Butyl acetate	838	-	-	0.01 (0.01)	-	-	-	0.01 (0.01)	-	-	-	-	0.01 (0.01)
50	[Ethyl 2-methylbutyrate]	865	-	-	-	-	-	-	-	-	-	-	-	0.01 (0.01)
51	3-Hexen-1-ol	874	-	-	-	-	-	0.03 (0.03)	-	0.03 (0.03)	-	-	0.04 (0.04)	0.04 (0.04)
52	1-Hexanol	887	0.02 (0.02)	0.03 (0.03)	-	0.05 (0.04)	0.01 (0.01)	-	0.03 (0.03)	0.02 (0.02)	0.01 (0.01)	-	0.01 (0.01)	0.01 (0.01)
53	Nonane	900	-	-	-	-	-	-	-	-	-	-	-	-
54	Pentyl acetate	930	-	-	-	-	-	-	-	-	0.01 (0.01)	-	-	-
55	Hexyl formate	957	-	0.5 (0.5)	-	-	-	-	0.1 (0.1)	-	-	-	-	-
56	Decane	1000	-	0.08 (0.06)	-	-	-	-	-	-	-	-	-	-
57	(Z)-3-Hexenyl acetate *	1019	0.04 (0.04)	-	0.11 (0.03)	0.04 (0.02)	0.12 (0.04)	-	0.08 (0.04)	0.2 (0.2)	0.01 (0.01)	0.01 (0.01)	0.2 (0.1)	0.3 (0.2)
58	Hexyl Acetate	1027	0.06 (0.03)	0.02 (0.02)	0.04 (0.02)	0.12 (0.07)	0.04 (0.02)	-	0.05 (0.05)	0.07 (0.04)	0.01 (0.01)	0.01 (0.01)	0.01 (0.01)	0.05 (0.02)
59	(E)-2-Hexenyl acetate	1037	-	-	-	0.04 (0.04)	-	-	0.03 (0.03)	0.09 (0.07)	0.01 (0.01)	-	-	0.03 (0.03)
60	Undecane	1100	-	-	-	-	-	-	-	0.05 (0.04)	-	-	-	-
61	Nonanal	1128	0.03 (0.03)	0.01 (0.01)	-	0.03 (0.02)	-	-	0.01 (0.01)	-	-	0.02 (0.02)	0.01 (0.01)	-
62	[3-Hexenylbutanoate]	1197	-	-	0.02 (0.01)	-	-	-	-	-	-	-	-	0.02 (0.02)
63	Dodecane *	1200	-	0.01 (0.01)	-	0.09 (0.03)	-	-	-	-	-	0.01 (0.01)	0.02 (0.02)	-
64	[(E)-3-Hexenyl-2-methylbutanoate]	1242	-	-	-	-	-	-	-	-	-	-	-	0.01 (0.01)
65	Tridecane *	1300	-	-	-	0.13 (0.06)	-	-	-	0.05 (0.05)	-	-	-	-
66	2-Undecanone *	1314	-	0.4 (0.2)	-	0.2 (0.1)	-	0.12 (0.04)	-	-	-	0.15 (0.08)	-	-
67	[3-Hydroxy-2,2-dimethylhexylbutanoate]	1397	-	-	-	0.09 (0.05)	-	-	-	-	-	-	-	-
68	Tetradecane	1400	-	-	-	-	-	-	-	-	-	0.03 (0.03)	-	-
69	Pentadecane *	1500	-	0.13 (0.05)	-	0.11 (0.07)	-	0.2 (0.1)	0.01 (0.01)	0.02 (0.01)	0.01 (0.01)	0.4 (0.3)	0.02 (0.02)	0.02 (0.02)
70	2-Tridecanone *	1516	-	3 (1)	-	4 (2)	-	1.8 (0.8)	-	-	-	3 (1)	-	-
71	[Dodecenyl acetate] *	1692	-	0.2 (0.1)	-	0.2 (0.1)	-	0.3 (0.1)	-	-	-	0.4 (0.2)	-	-
72	Heptadecane	1700	-	-	-	-	-	-	-	-	-	-	-	-
73	2-Pentadecanone *	1718	-	8 (4)	-	9 (4)	-	6 (3)	-	-	-	12 (6)	-	-
74	2-Heptadecanone *	1922	0.16 (0.06)	1.5 (0.6)	-	3 (1)	0.7 (0.3)	0.7 (0.2)	0.05 (0.03)	0.05 (0.03)	-	1.2 (0.5)	0.13 (0.07)	0.07 (0.02)
75	Heneicosane	2100	-	-	-	-	-	-	-	0.01 (0.01)	-	-	-	-
***Yeast Associated Compounds***			***4 (2)***	***9 (6)***	***0.9 (0.5)***	***3 (1)***	***9 (2)***	***16 (11)***	***2 (1)***	***6 (4)***	***18 (8)***	***12 (7)***	***12 (9)***	***1.8 (0.9)***
76	Ethanol	426	4 (2)	9 (6)	0.9 (0.5)	3 (1)	9 (2)	15 (11)	2 (1)	6 (3)	17 (7)	12 (7)	12 (9)	1.8 (0.9)
77	Ethyl acetate	615	0.01 (0.01)	-	0.01 (0.01)	-	0.01 (0.01)	0.04 (0.03)	0.08 (0.08)	0.1 (0.1)	0.3 (0.2)	0.01 (0.01)	0.09 (0.09)	0.04 (0.03)
78	2-Methyl-1-propanol	624	-	-	-	0.1 (0.1)	-	-	-	-	-	-	-	-
79	Acetic acid	639	-	-	-	-	-	-	-	-	-	-	-	-
80	3-Hydroxy-2-butanone	748	-	-	-	0.1 (0.1)	-	0.2 (0.2)	-	-	0.02 (0.02)	-	-	-
81	Ethyl butanoate *	820	-	-	0.01 (0.01)	-	-	0.3 (0.2)	0.01 (0.01)	-	0.5 (0.2)	-	-	-
***Unidentified***			***0.2 (0.1)***	***1.3 (0.7)***	***0.16 (0.02)***	***1.2 (0.4)***	***0.15 (0.05)***	***1.3 (0.4)***	***0.7 (0.2)***	***0.4 (0.2)***	***0.17 (0.08)***	***0.5 (0.2)***	***0.3 (0.1)***	***0.42 (0.06)***

* significantly different relative amounts in L and S flowers.

**Table 2:** Major (>1%) floral scent compounds in *G. sempervirens* in order of relative amounts. Compounds with significantly different relative amounts in L verses S flowers (*p* < 0.05) are indicated with an asterisk.

#	Compound	Percent Occurrence (%)		Mean Relative Amount (%)	
		L Style (N = 38)	S Style (N = 36)	L Style (N = 38)	S Style (N = 36)
	**Benzenoids**			**77**	**52**
2	Benzaldehyde *	100	100	55	37
20	Benzyl benzoate *	100	75	8.4	5.3
13	4-Anisaldehyde *	100	64	6.1	3.6
5	Acetophenone	87	89	2.6	2.5
3	4-Methylanisole	95	64	2.0	1.3
4	Benzyl alcohol *	84	56	2.0	1.1
	**Terpenoids**			**14**	**30**
29	(*Z*)-α-Ocimene *	32	58	0.02	16
38	α-Farnesene *	74	44	5.4	4.0
39	β-Ionone	26	22	3.1	2.6
32	Dihydro-β-ionone *	66	81	0.8	3.1
34	α-Ionone *	97	61	1.8	1.3
21	α-Pinene	68	67	1.0	0.3
	**Fatty Acid Derivatives**			**0.5**	**9.7**
73	2-Pentadecanone *	0	42	-	5.8
70	2-Tridecanone *	0	33	-	1.8
74	2-Heptadecanone *	42	75	0.2	1.1
	**Yeast Compounds**			**7.8**	**8.1**
76	Ethanol	92	94	7.7	8.0
	**Total Peak Area (counts)**			**49.6 ± 4.9 million**	**36.8 ± 4.9 million**

* significantly different relative amounts in L and S flowers.

**Table 3:** Locations of wild and cultivated populations of *Gelsemium sempervirens* sampled.

Label	Population Type	Population	Location	GPS Location	# of Flowers and Style Morph
C1	Cultivated	USC Lancaster Arbor	Lancaster, SC	34°44′14.1” N 80°47′01.3” W	3 L 6 S
C2	Cultivated	Waterstone Neighborhood	Fort Mill, SC	35°03′04.0” N 80°58′53.2” W	6 L 6 S
C3	Cultivated	Baxter Village	Fort Mill, SC	35°01′44.7” N 80°58′00.6” W	8 L 8 S
W1	Wild	USC Lancaster Nature Trail	Lancaster, SC	34°44′15.6” N 80°47′00.4” W	8 L 6 S
W2	Wild	Andrew Jackson State Park	Lancaster, SC	34°50′37.6” N 80°48′29.1” W	7 L 6 S
W3	Wild	Anne Springs Close Greenway	Fort Mill, SC	35°02′07.6” N 80°55′01.1” W	6 L 8 S

# represents the number of flowers sampled.
